# Editorial: Toll-like receptor expression in transformed cells: role in tumor development and cancer therapies

**DOI:** 10.3389/fimmu.2024.1478431

**Published:** 2024-08-22

**Authors:** Fabian Benencia, Laura D. Alaniz, Kelly D. McCall

**Affiliations:** ^1^ Molecular and Cellular Biology Program, Ohio University College of Arts & Sciences, Athens, OH, United States; ^2^ Department of Biomedical Sciences, Ohio University Heritage College of Osteopathic Medicine, Athens, OH, United States; ^3^ Diabetes Institute, Ohio University Heritage College of Osteopathic Medicine, Athens, OH, United States; ^4^ Biomedical Engineering Program, Russ College of Engineering and Technology, Ohio University, Athens, OH, United States; ^5^ Laboratorio de Microambiente Tumoral, CIBA, UNNOBA. CIT NOBA (UNNOBA-UNSADA-CONICET), Junín, Argentina; ^6^ Department of Biological Sciences, Ohio University College of Arts & Sciences, Athens, OH, United States; ^7^ Department of Specialty Medicine, Ohio University Heritage College of Osteopathic Medicine, Athens, OH, United States

**Keywords:** toll-like receptors, tumor microenvironment (TME), cancer immune cell therapy, B lymphocytes, PAMP (pathogen-associated molecular pattern), DAMP (damage-associated molecular pattern)

In a tumor setting, the immune system can function as a double-edged sword, helping control tumor development or participating in cancer progression. Indeed, chronic inflammation has been considered a factor linked to cancer progression (1). Pathogen recognition receptors (PRRs) were originally recognized as microbial detectors harbored by immune cells. Toll-like receptors (TLRs) constitute a family of membrane-bound PRRs which can recognize pathogen-associated molecular patterns (PAMPs) carried by bacteria (e.g., LPS which interacts with TLR-4), viruses (e.g., CpG DNA interacting with TLR-9; ssRNA, ligand for TLR-7; or dsRNA generated during infection which can activate TLR-3), and fungi (e.g., beta glucans interacting with TLR-2). PRRs can also recognize products generated by stressed cells or damaged tissues, named damage-associated molecular patterns (DAMPs) and therefore participate in the biology of sterile inflammation. For example, non-infectious molecules such as hyaluronan, an extracellular matrix component of the tumor microenvironment that has been recognized as a tumor promoter and a therapeutic target for several cancers, such as breast cancer (2) can activate TLR-2, -4 and -6 (3).

In addition to being expressed by immune cells, TLRs are also expressed by tumor cells, among others, pancreatic, breast, and ovarian cancer cells, and have significant effects on the inflammatory profile of the tumor milieu (4). This also includes expression of TLRs by hematological tumors such as leukemias or lymphomas (5).

The effects of TLR activation on tumor cells varies depending on the specific TLR and tumor type under consideration (4). Foundational studies on the role of TLR-3 signaling in tumor cells were conducted in thyroid cancer by McCall et al. (6). They demonstrated that functional TLR-3 was present in human papillary thyroid cancer (PTC) cell lines at high basal levels consistent with its overexpression in PTC cells *in vivo* and that inhibition of its expression and signaling decreased tumor cell proliferation and migration (6); follow-up studies revealed similar findings in other types of human cancers (7–9). In addition, studies from the Benencia lab demonstrate that TLR-3 agonists (dsRNA analogs poly [I:C] and poly [A:U]) act on breast and ovarian tumor cells by inducing inflammatory cytokines such as IL6 and CCL5 (10, 11).

Expression of TLRs by tumor cells and their activation has been associated with tumor progression in several types of cancer (4). In this scenario, the use of TLR inhibitors as a therapeutic approach to fight certain types of cancer may enhance patient survival. Inhibition of some of these molecules in cancers cells can lead to a decrease in tumor growth and can be used in combinatorial therapies with other anticancer agents. On the contrary, studies have also described an association between TLR expression and better outcomes in some tumor settings which would support the use of TLR agonists for cancer therapy (4). Altogether, this conflicting information highlights the need to further investigate TLR biology to unravel the role of these molecules in tumor development and design novel therapeutic approaches against cancer.

This Research Topic harbors two reviews and four original research articles that summarize current information on the role of TLRs in tumor development and immunotherapies, and presents recent studies that increase our understanding of the relevance of these molecules in tumor settings.

In a review article, Zhou et al. provide a summary of the role of TLRs in breast cancer development and the possibility of targeting these molecules for immunotherapeutic approaches. They describe both pro-tumorigenic and anti-tumorigenic roles of TLRs in breast cancer, specifically, TLR-2,-3, -4, -5, -7 and 9. They describe the expression of these molecules both in tumor cells or other cells of the tumor microenvironment, such as immune cells and endothelial cells and discuss the use of TLR agonists for cancer immunotherapy.

In a second review article, Qi et al. comprehensively summarize the current knowledge of TLR expression and regulation in HPV-associated cancers, such as cervical carcinoma, skin cancer, and head and neck cancer. They describe studies using TLR agonists alone, or in combination with vaccines or checkpoint inhibitors as therapeutic approaches for HPV-associated cancers.

A research article from Tryggestat et al. investigated the role of TLR signaling in promoting the expression of protumor molecules in multiple myeloma cells. In this study, they were able to determine that TLR-4 and -9 activation in multiple myeloma cells increased expression of pro-survival genes, provided resistance to proteosome inhibitor therapies, and reduced the expression of therapeutic targets on the cell surface.

A research article from Zhang et al. described a therapeutic approach for reprogramming myeloid cells in the tumor microenvironment by delivering a TLR7 agonist to tumor-associated macrophages which express active folate receptor beta, in contrast to other macrophages in the body. This approach highlights the need to specifically target cell populations on the tumor microenvironment with TLR agonists to prevent toxicities or undesired cell activation.

A research study from Kawata et al. focused on defining the effect of inhibiting Bruton’s tyrosine kinase (BTK) on the septic shock induced by LPS, a TLR4 ligand. They studied BTK-mediated TLR4 signaling in B cells and defined the effect of blocking BTK on IL6 production by B cells. This study highlights the role of different signaling molecules associated with TLR activation and can help to further investigate their role in B cell malignancies.

Monti et al. study the effect of melanoma-suppressive cytokines on TLR-7 and -9 activation in plasmacytoid dendritic cells (pDCs), showing that tumor-derived cytokines reduce pDC responsiveness to TLR-7 and -9 stimulation, shifting them towards a tolerogenic state. This finding is relevant for therapeutic strategies using TLR agonists in melanoma.

In conclusion, this Research Topic illustrates the complex role of TLRs in tumor biology, affecting cancer cells and other cells in the tumor microenvironment. It also highlights the need for refined therapeutic approaches to leverage TLR activation in cancer therapy while avoiding toxic effects or tumor progression.
